# COGNABILITY: AN ECOLOGICAL THEORY OF NEIGHBORHOODS AND COGNITIVE AGING

**DOI:** 10.1093/geroni/igac059.027

**Published:** 2022-12-20

**Authors:** Jessica Finlay, Esposito Michael, Kenneth Langa, Suzanne Judd, Philippa Clarke

**Affiliations:** University of Michigan, Ann Arbor, Michigan, United States; Washington University in St. Louis, St. Louis, Missouri, United States; University of Michigan, Ann Arbor, Michigan, United States; University of alabama at Birmingham, Birmingham, Alabama, United States; University of Michigan, Ann Arbor, Michigan, United States

## Abstract

This paper presents a new theoretical concept, Cognability, which aims to conceptualize how supportive an area is to cognitive health among aging residents. Cognability incorporates a both positive and negative neighborhood features related to physical activity, social interaction and cognitive stimulation in later life. We analyzed data from the Reasons for Geographic and Racial Differences in Stroke Study, a national sample of older Black and white US adults (n=21,151; mean age at assessment=67; data collected 2006–2017). Generalized additive multilevel models examined how cognitive function varied by neighborhood features. Access to civic and social organizations, recreation centers, fast-food and coffee establishments, arts centers, museums, and highways were significantly associated with cognitive function. Race-, gender-, and education-specific models did not yield substantial improvements to the full-model. Cognability advances ecological theories of aging through an innovative “whole neighborhood” approach. Findings may inform community interventions and policy to support healthy aging in place.

